# Invasive bacterial disease trends and characterization of group B streptococcal isolates among young infants in southern Mozambique, 2001–2015

**DOI:** 10.1371/journal.pone.0191193

**Published:** 2018-01-19

**Authors:** Betuel Sigaúque, Miwako Kobayashi, Delfino Vubil, Ariel Nhacolo, Alberto Chaúque, Benild Moaine, Sérgio Massora, Inácio Mandomando, Tacilta Nhampossa, Quique Bassat, Fabiana Pimenta, Clara Menéndez, Maria da Gloria Carvalho, Eusebio Macete, Stephanie J. Schrag

**Affiliations:** 1 Centro de Investigação em Saúde de Manhiça, Maputo, Mozambique; 2 John Snow Inc. (JSI) on the Maternal and Child Survival Program–MCSP (USAID Grantee), Maputo, Mozambique; 3 Division of Bacterial Diseases, National Center for Immunization and Respiratory Diseases, Centers for Disease Control and Prevention, Atlanta, United States of America; 4 ISGlobal, Barcelona Center for International Health Research, and Hospital Clinic-Universitat de Barcelona, Barcelona, Spain; 5 Consorcio de Investigación Biomédica en Red de Epidemiología y Salud Pública, Barcelona, Spain; Universidade de Lisboa Faculdade de Medicina, PORTUGAL

## Abstract

**Background:**

Maternal group B streptococcal (GBS) vaccines under development hold promise to prevent GBS disease in young infants. Sub-Saharan Africa has the highest estimated disease burden, although data on incidence and circulating strains are limited. We described invasive bacterial disease (IBD) trends among infants <90 days in rural Mozambique during 2001–2015, with a focus on GBS epidemiology and strain characteristics.

**Methods:**

Community-level birth and mortality data were obtained from Manhiça’s demographic surveillance system. IBD cases were captured through ongoing surveillance at Manhiça district hospital. Stored GBS isolates from cases underwent serotyping by multiplex PCR, antimicrobial susceptibility testing, and whole genome sequencing.

**Results:**

There were 437 IBD cases, including 57 GBS cases. Significant declines in overall IBD, neonatal mortality, and stillbirth rates were observed (P<0.0001), but not for GBS (P = 0.17). In 2015, GBS was the leading cause of young infant IBD (2.7 per 1,000 live births). Among 35 GBS isolates available for testing, 31 (88.6%) were highly related serotype III isolates within multilocus sequence types (STs) 17 (68.6%) or 109 (20.0%). All seven ST109 isolates (21.9%) had elevated minimum inhibitory concentration (MIC) to penicillin (≥0.12 μg/mL) associated with penicillin-binding protein (PBP) 2x substitution G398A. Epidemiologic and molecular data suggest this is a well-established clone.

**Conclusion:**

A notable young infant GBS disease burden persisted despite improvements in overall maternal and neonatal health. We report an established strain with *pbp2x* point mutation, a first-step mutation associated with reduced penicillin susceptibility within a well-known virulent lineage in rural Mozambique. Our findings further underscores the need for non-antibiotic GBS prevention strategies.

## Introduction

In 2013, an estimated 6.3 million children aged <5 years died globally; more than half of these deaths were due to infectious causes[1]. Causes with the slowest reduction in under-five mortality rates included congenital abnormalities, preterm birth complications, and neonatal sepsis[1]. In countries with strong invasive bacterial disease (IBD) surveillance, group B *Streptococcus* (GBS) is a leading cause of sepsis and bacterial meningitis in the first 90 days of life[2–4] with a notable portion of cases presenting on the day of birth[5, 6]. GBS has also been associated with stillbirths[7, 8] and premature births[9]. A systematic review estimated that the global incidence of GBS disease in young infants (<90 days) was 0.53 per 1,000 live births and highest in sub-Saharan Africa (1.21 per 1,000 live births)[10]. Estimated case fatality was also the highest in this region[10].

In resource-rich countries, intrapartum antibiotic prophylaxis (IAP) has been used to prevent neonatal GBS disease since the 1980s, with success in reducing early-onset (disease onset during days 0–6) GBS disease [11–13]. However, the impact has been variable in different settings adopting different IAP strategies [14, 15], and IAP has not impacted late-onset (disease onset during days 7–89) disease[16]. In addition, IAP may not be feasible to implement in resource-limited settings. In recent years, there has been increasing global interest in maternal vaccination during pregnancy as a strategy to prevent disease with significant burden among neonates and infants. The World Health Organization (WHO) Product Development for Vaccines Advisory Committee (PDVAC) identified GBS as a priority pathogen[17]. Current GBS vaccines under development use capsular polysaccharides and surface proteins as the main vaccine targets, and some have completed phase II clinical trials[18–20].

A better understanding of the GBS disease burden and bacteriologic characteristics in low-income, high-burden settings will help inform GBS vaccine decision making[17]. However, current disease burden data from low- and middle-income countries (LMIC) are sparse[10]. Limited access to health care, large proportion of births outside healthcare facilities, and lack of diagnostic capacity likely contribute to underestimation of the disease burden among young infants in LMIC[21]. Here, we aimed to describe IBD trends from 2001–2015 among infants aged <90 days in a rural district in southern Mozambique and characterize clinical and microbiological features of early- and late-onset GBS disease.

## Materials and methods

### Manhiça demographic surveillance system

The Manhiça Health Research Center (Centro de Investigação em Saúde de Manhiça, CISM) is located in a rural area 80 km north of Maputo, Mozambique. Since 1996, CISM has run a continuous demographic surveillance system (DSS), which captures births, deaths, causes of deaths, maternity, in and out migration, and pregnancies from a defined catchment area[22, 23]. Causes of deaths in the area are obtained through verbal autopsy using methods based on the WHO model. There is perennial malaria transmission in the area, mostly due to *Plasmodium falciparum[24]* and high HIV prevalence (23.6%) among pregnant women[25]. Vaccines against *Haemophilus influenzae* type b (Hib) and the 10-valent pneumococcal conjugate vaccine (PCV10, three doses at 2, 3, and 4 months) were introduced into the national immunization program in 2009 and 2013, respectively[26]. The DSS catchment area expanded in phases, starting with the town of Manhiça in 1996 (100 km^2^, approximately 34,000 residents), the addition of surrounding neighborhoods in 2002 and 2005, and expansion to the entire Manhiça district in 2014 (2,380km^2^, population size of 175,000). The DSS catchment contains 12 health centers and two referral district hospitals; most patients requiring admission are referred to the Manhiça district hospital (MDH). Young infants with illness requiring intensive care, including those <28 weeks of gestation or very low birth weight (<1,500g), are transferred to the national referral hospital (Maputo Central Hospital) in Maputo.

### Invasive bacterial disease surveillance

Since January 1997, CISM and MDH have jointly operated pediatric IBD surveillance, including capture of young infant infections, through round-the-clock surveillance of pediatric outpatient department visits and hospital admissions. Trained healthcare workers use standardized forms to record physical examination findings and clinical signs reported by the caregiver upon admission[23, 27]. A single venous blood specimen for bacterial culture is collected from all young infants before hospital admission. Cerebrospinal fluid (CSF) collection is recommended for any neonate (age ≤28 days) or older infants with a reported history of convulsions, agitation, unconsciousness, or depressed or tense fontanelle on examination. Recovered isolates are stored in a biobank. Additional laboratory test results (e.g., blood glucose, blood smears), treatment details, discharge diagnoses, and hospital outcome are recorded. Forms were double entered in FoxPro (version 2.6, Microsoft Corporation, Redmond, WA, USA) at CISM and discrepancies in data entry were resolved by referring to the original forms. IBD surveillance data are linked to the DSS records.

### Laboratory procedures

Approximately 1 ml of whole blood was immediately inoculated into one pediatric blood culture bottle (Pedibact®; Becton-Dickinson, Franklin Lakes, NJ, USA), which was incubated in an automatic BACTEC® 9050 system (Becton-Dickinson) for 5 days. Blood culture bottles with growth detected were subcultured onto blood agar and incubated overnight at 37°C in 5% CO_2_ for 18–24 hours. CSF samples first underwent cytological examination and Gram stain, and were subsequently cultured onto blood agar, chocolate agar and thioglycolate broth media for 24 hours and, if negative, up to 5 days at 37°C in an atmosphere of 5% CO2. Thioglycolate broths with turbidity were stained and subcultured on blood agar media and incubated for 48 hours. Bacterial isolates were identified according to standard microbiologic procedures as described previously[27]. Typical colonies of GBS were identified by beta-hemolysis or non-hemolysis (nonhemolytic variant), Gram stain compatible with *Streptococcus* spp., negative catalase reaction, bacitracin resistance, and detection of the Lancefield group B antigens by a rapid latex agglutination test (Bio RAD PASTOREX^TM^ STREP). GBS isolates were sent to the *Streptococcus* Laboratory at the U.S. Centers for Disease Control and Prevention (CDC), Atlanta, for serotyping by multiplex PCR, antimicrobial susceptibility testing by broth microdilution using Clinical and Laboratory Standards Institute minimum inhibitory concentration (MIC) breakpoints[28], and whole genome sequencing (WGS). If both blood and CSF isolates were available from the same child, the CSF isolate was selected for WGS. WGS procedures for serotype deduction, deduction of antimicrobial susceptibility profiles, and MLST are described in detail elsewhere[29, 30]. For determination of presence or absence of certain surface proteins implicated in adhesion, carriage, and disease, we employed sequence queries for the CC17-associated virulence factor (hypervirulent GBS adhesin, HvgA), three different pili (PI1, PI2a and PI2b-1), four different Alpha C family proteins (Alp1, Alp2-3, Alpha, Rib), and the two serine rich repeat proteins (SRR1 and SRR2) (S1 Table). Our designated Alp2-3, a close homolog of the group A *Streptococcus* R28 antigen[31], is representative of one of four mutually exclusive, distinct Alpha C family genetic determinants detected through our pipeline. PI2a1 and PI2a2 are subclasses of the backbone protein subunit PI2a [32]. The bioinformatics pipeline is described athttps://github.com/BenJamesMetcalf/GBS_Scripts_Reference. To perform single nucleotide polymorphism analysis, paired-end fastq files were trimmed with Cutadapt v 1.8.1 and draft genome assemblies were constructed using VelvetOptimiser v 2.2.5 with an optimal kmer value calculated by VelvetK. Core genome SNP identification and alignment were carried out using kSNP3.0[33].

### Definitions

Microbiologically-confirmed IBD was defined as a positive blood or CSF culture from a young infant from the DSS catchment area admitted to MDH. Coagulase-negative staphylococci, group viridans streptococci, and gram-positive bacilli (e.g., *Corynebacterium* spp., *Bacillus* spp.) were considered contaminants. Early-onset disease (EOD) was defined as IBD in children aged <7 days, and late-onset disease (LOD) as IBD in children aged 7–89 days. Possible serious bacterial infection (PSBI) was defined by any one of the following signs: poor feeding, history of convulsions according to the caregiver’s report, healthcare worker observation of fast breathing (defined as respiratory rate of ≥60 per minute in a child 0–59 days and ≥50 per minute in a child 60–89 days), chest indrawing, fever (≥38.0°C), low body temperature (35.5°C<), or unconsciousness.

### Statistical analysis

We calculated annual IBD incidence rate, neonatal mortality rate, and admission rate using the number of live births per year within the DSS catchment area as the denominator. Annual stillbirth rates were calculated using the number of births (live births and stillbirths) as the denominator. Due to time lags in finalization of DSS data, neonatal mortality rate was evaluated from 2001–2013 and verbal autopsy data from 2001–2011. Proportions were compared using chi-square or Fisher’s exact tests. Trends of rates were assessed using Poisson regression and trends of proportion were assessed using the Cochran-Armitage trend test. All analyses were conducted in SAS 9.4 (SAS Institute, Inc., Cary, North Carolina).

### Ethics

This study retrospectively assessed data collected in the context of routine surveillance. As part of the DSS and IBD surveillance procedure, written consent is obtained from all heads of households and adult participants to use their and their children’s data (if age <18 years) for research conducted by CISM. The IBD surveillance system was reviewed and approved by the Mozambican National Bioethics Committee and institutional review boards of Hospital Clinic of Barcelona, Spain, the U.S. CDC and the University of Maryland, School of Medicine.

## Results

**Fig 1:**
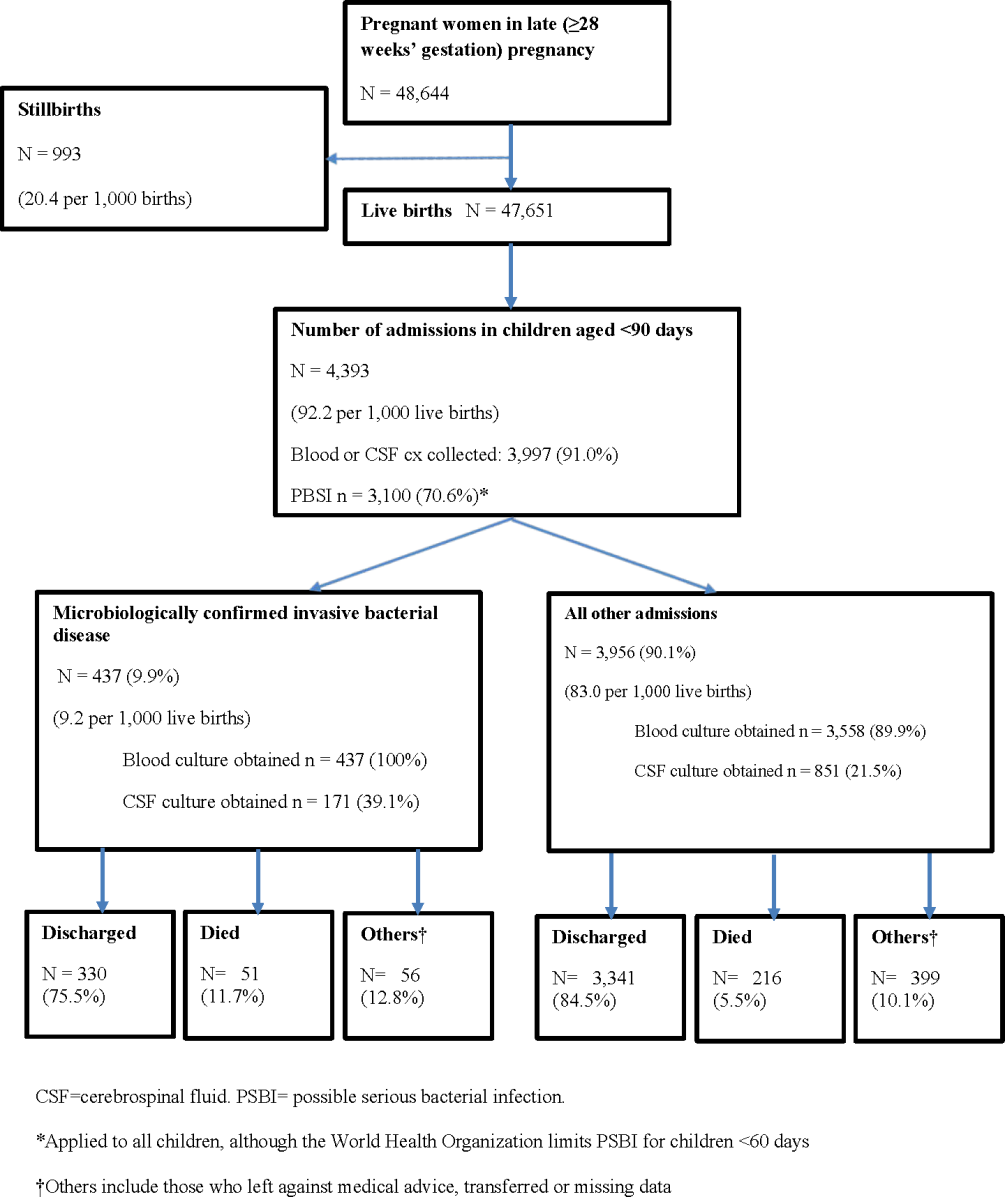
Summary of young infants aged <90 days admitted for invasive bacterial disease, Manhiça demographic and health surveillance system, Mozambique, 2001–2015.

**Fig 2:**
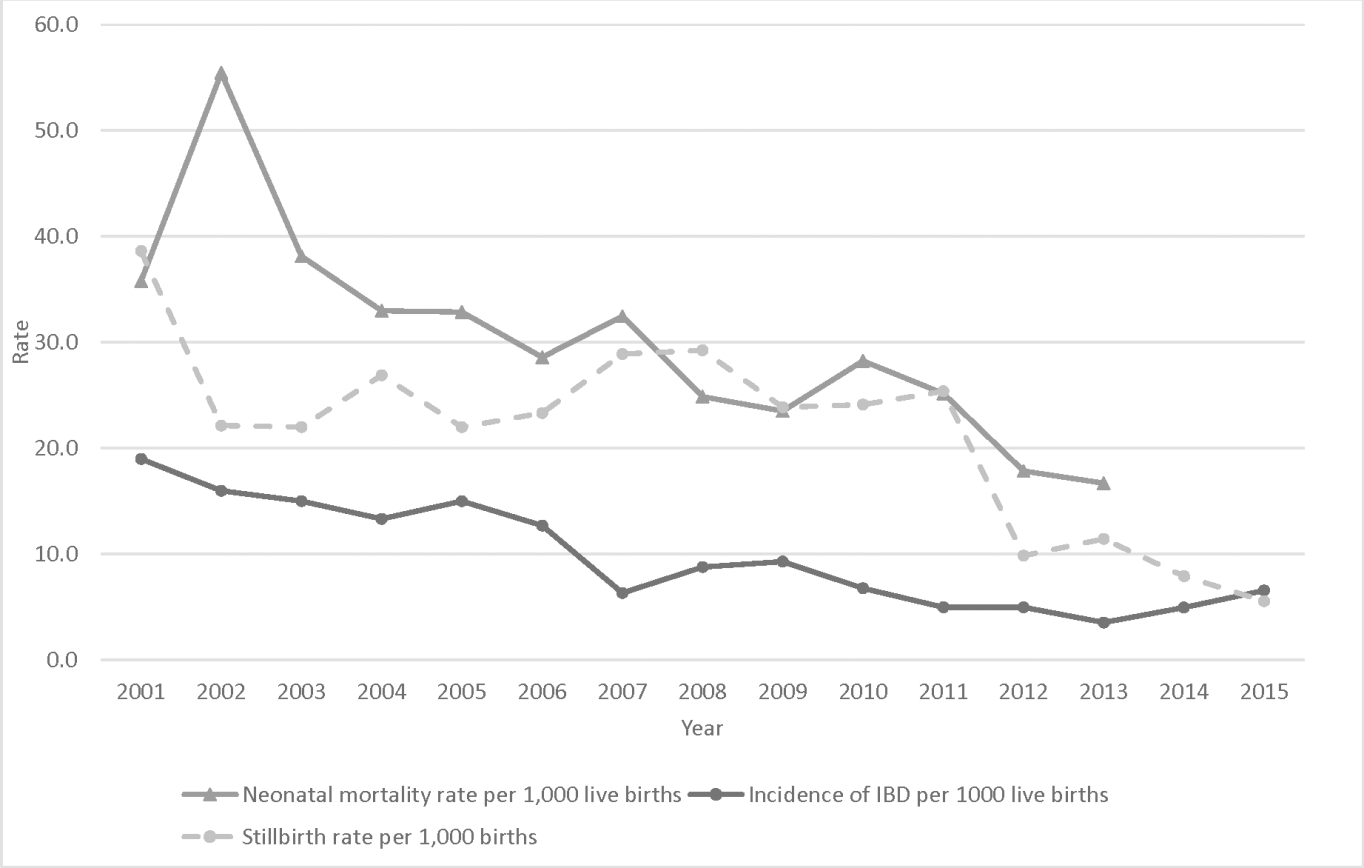
Trends of invasive bacterial disease and neonatal mortality rates (per 1,000 live births) during 2001–2015 and stillbirth rate (per 1,000 births) during 2001–2013, Manhiça, Mozambique.

During 2001–2015, a total of 47,651 live births and 993 stillbirths were reported within the DSS catchment (Fig 1, S2 Table). The number of annual live births tripled from 2001 (1,370) to 2015 (4,120), reflecting the DSS expansion. In 2014–15 (years where birth location was captured) most (93.7%) births occurred at health facilities. Both stillbirth and neonatal mortality rates declined during the study period (stillbirths: 38.6 per 1,000 births in 2001 to 5.6 per 1,000 births in 2015, *P*<0.0001; neonatal mortality: 35.8 per 1,000 live births in 2001 to 16.7 per 1,000 live births in 2013, *P*<0.0001) (Fig 2). Among infants <90 days, 2,257 deaths were reported. Based on verbal autopsy records (2001–2011), 53.6% of young infant deaths occurred at a health facility (*P* = 0.66 for trend); leading causes of death were respiratory and cardiovascular diseases specific to the perinatal period (19.8%), disorders related to length of gestation and fetal growth (14.5%), and infection specific to the perinatal period (12.9%) (S3 Table).

A total of 4,393 admissions of children aged <90 days was recorded at MDH (range: 206–388 per year). The admission rate decreased from 151 per 1,000 live births in 2001 to 59 per 1,000 live births in 2015 (*P*<0.0001). Of all admissions, 91% (3,997/4,393) had blood or CSF culture collected (78.5% for day 0–2 infants vs. 90.7% for day ≥3 infants; *P*<0.0001) and 9.9% (437/4,393) had IBD (EOD: 23.1%; LOD: 76.9%) (Fig 1). Median age of onset of infants with IBD was 15 days (interquartile range [IQR] 7–29) (S1 Fig); 13.1% had been seen at another healthcare facility before admission and 76.0% had signs consistent with PSBI **(**Table 1**)**. Fast breathing (51.4%) and fever (37.0%) were the most common signs (S5 Table). Of the 354 with information on antibiotic administration, most (98.6%) infants with IBD received antibiotic treatment, primarily aminoglycosides (gentamicin) (79.9%) and ampicillin or amoxicillin (73.6%). Of those with IBD, 51 (11.8%) died during the hospital stay, compared to 216 of 3956 (5.5%) admitted infants without IBD (*P*<0.0001) **(**Fig 1**)**.

**Table 1:** Descriptive characteristics of young infants days 0–89 of age with IBD, Manhiça demographic and health surveillance system, Mozambique, 2001–2013.

Characteristics	All IBD N = 437 No (%)	All GBS N = 57 No (%)	GBS	
			EOD N = 19 no (%)	LOD N = 38 no (%)
**Sex**				
Male	239 (54.7%)	30 (52.6%)	10 (52.6%)	20 (52.6%)
**Median age in days (IQR)**	15 (7–29)	10 (4–15)	0 (0–4)	13 (10–17)
**Median weight upon admission, kg (IQR)**^**1**^	3.3 (2.8–3.8)	3.0 (2.7–3.4)	2.9 (2.6–3.1)	3.1 (2.8–3.5)
**Child is breastfed**^**2**^	320 (90.4%)	44 (91.7%)	16 (94.1%)	28 (90.3%)
**Caregiver previously sought care for child’s condition**^**3**^				
Yes	57 (13.1%)	4 (7.0%)	2 (10.5%)	2 (5.3%)
No	377 (86.7%)	53 (93.0%)	17 (89.5%)	36 (94.7%)
**PSBI present upon admission**^4^				
Yes	322 (76.0%)	47 (82.5%)	17 (89.5%)	30 (79.0%)
No	105 (24.0%)	10 (17.5%)	2 (10.5%)	8 (21.1%)
**Clinical signs reported by the caregiver**				
History of convulsions^5^	18 (4.2%)	3 (5.3%)	2 (10.5%)	1 (2.6%)
Cough^6^	182 (41.7%)	17 (29.8%)	2 (10.5%)	15 (39.5%)
Difficult breathing^7^	150 (34.6%)	22 (39.3%)	10 (52.6%)	12 (32.4%)
Unable to drink or breastfeed^8^	82 (18.9%)	17 (29.8%)	10 (52.6%)	7 (18.4%)
Diarrhea	42 (9.6%)	1 (1.8%)	1 (5.3%)	0
Vomiting	42 (9.6%)	3 (5.3%)	1 (5.3%)	2 (5.3)
**Type of specimen**				
Blood only	266 (60.9%)	33 (57.9%)	12 (63.2%)	21 (55.3%)
Blood and CSF	171 (39.3%)	24 (42.1%)	7 (36.8%)	17 (44.7%)
Meets criteria for CSF culture	348 (79.6%)	56 (98.3%)	19 (100%)	37 (97.4%)
CSF collected among those who have indications for collection^9^	171 (49.1%)	24 (42.9%)	7 (36.8%)	17 (45.9%)
CSF culture positive	53/171 (31.0%)	15/24 (62.5%)	5/7 (71.4%)	10/17 (58.8%)
**Antibiotic treatment given**^**10**^	349 (98.6%)	46 (95.8%)	16 (94.1%)	30 (96.8%)
Penicillin	57/349 (16.3%)	4/46 (8.7%)	1/16 (6.3%)	3/30 (10.0%)
Cotrimoxazole	25/349 (7.2%)	0	0	0
Chloramphenicol	15/349 (4.3%)	0	0	0
Gentamicin	279/349 (79.9%)	33/46 (71.7%)	10/16 (62.5%)	23/30 (76.7%)
Ampicillin/Amoxicillin	257/349 (73.6%)	36/46 (78.3%)	11/16 (68.8%)	25/30 (83.3%)
Erythromycin	8/348 (2.3%)	0	0	0
Cephalosporins	90/348 (25.9%)	15/46 (32.6%)	8/16 (50.0%)	7/30 (23.3%)
**Outcome of hospital stay**				
Discharged	330 (75.5%)	48 (84.2%)	12 (63.2%)	36 (94.7%)
Died	51 (11.7%)	7 (12.3%)	6 (31.6%)	1 (2.6%)
Others^11^	56 12.8%)	2 (3.5%)	1 (5.3%)	1 (2.6%)

CSF = cerebrospinal fluid. EOD = early-onset disease. GBS = group B *streptococcus*. IBD = invasive bacterial disease. IQR = interquartile range. LOD = late-onset disease. PSBI = possible serious bacterial infection.

1.17 missing data

2. 83 were missing data, and the percentage was calculated excluding those with missing information (9 missing for GBS: EOD 2, LOD 7).

3. 3 were missing data (none among GBS cases), and the percentage was calculated excluding those with missing information.

4.Defined as presence of either: unable to drink or breastfeed, history of convulsions, fast breathing, chest indrawing, fever (≥38.0°C), low body temperature (35.5°C<), unconscious

5. 4 were missing data (none among GBS cases), and the percentage was calculated excluding those with missing information.

6. 1 was missing data (none among GBS cases), and the percentage was calculated excluding those with missing information.

7. 3 were missing data, including 1 LOD case. The percentage was calculated excluding those with missing information.

8. 2 were missing data (none among GBS cases), and the percentage was calculated excluding those with missing information.

9.Any neonate ≤28 days or infants 28 days with any of the following: depressed or tense fontanelle; convulsions reported by the caregiver; agitated or unconscious

10. 83 were missing data on antibiotic treatment, including 9 among GBS cases (2 EOD, 1 LOD). The percentage was calculated excluding those with missing information.

11.Transferred, left against medical advice, or missing data (4 cases, none among GBS cases).

**Fig 3:**
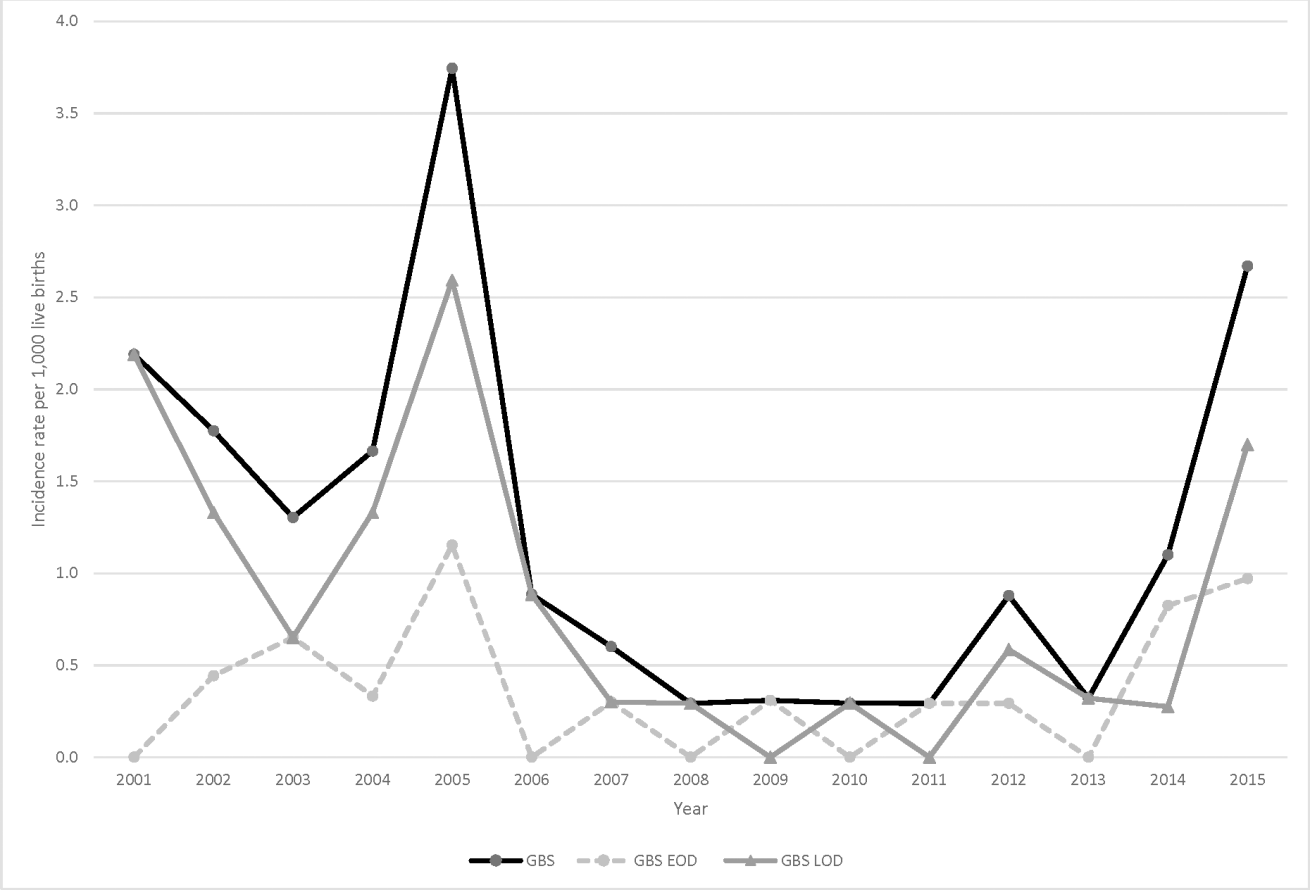
Trends of invasive GBS disease incidence rates in young infants (<90 days), Manhiça, 2001–2015.

Overall, *Staphylococcus aureus* was the most frequently isolated pathogen (n = 134, 2.8 per 1,000 live births), followed by GBS (n = 57, 1.2 per 1,000 live births) and *Streptococcus pneumoniae* (n = 54, 1.1 per 1,000 live births) (Table 2). The overall IBD incidence rate declined significantly during the study period (19.2 per 1,000 live births in 2001 to 6.6 per 1,000 live births in 2015; *P*<0.0001) (Fig 2). Incidence rates declined significantly for *S*. *aureus*, *S*. *pneumoniae*, group D *streptococcus*, *Escherichia coli*, and nontyphoidal *Salmonella* (Table 2, S2A and S2B Figs), but not for GBS (Table 2, Fig 3). GBS incidence rate peaked in 2005 at 3.7 per 1,000 live births, had a low, stable incidence from 2008–2011 (0.3 per 1,000 live births) and increased again to become the leading IBD pathogen in 2015 (2.7 per 1,000 live births) (S2A Fig).

**Table 2:** Bacterial isolations and incidence of invasive infections among young infants, Manhica demographic and health surveillance system, Mozambique, 2001–2015.

Species	Rate per 1000 live births^1^ (no.)					p value for trend over time^2^
	Overall	Day 0–2 of life	Day 3–6	Day 7–27	Day 28–89	
**Gram positive**	6·3 (307) ^3^	1·1 (52)	0·3 (13)	3·6 (175)	1·4 (67)	<0·001^4^
*Staphylococcus aureus*	2·8 (134)	0·4 (20)	0·1 (5)	1·9 (90)	0·4 (19)	<0·001^4^
Group B *streptococcus*	1·2 (57)	0·3 (14)	0·1 (5)	0·7 (35)	0·06 (3)	0·17
*Streptococcus pneumoniae*	1·1 (54)	0·06 (3)	0·02 (1)	0·3 (14)	0·7 (36)	0·001^4^
Group D *streptococcus*	0·7 (35)	0·3 (14)	0·04 (2)	0·3 (14)	0·1 (5)	<0·001^4^
Group A *streptococcus*	0·4 (18)	0	0	0·3 (16)	0·04 (2)	0·26
Others	0·2 (10)	0·02 (1)	0	0·1 (6)	0·06 (3)	0·10
**Gram negative**	2·8 (137) ^5^	0·6 (27)	0·3 (12)	1·0 (47)	1·0 (50)	<0·001^4^
*Escherichia coli*	0·7 (32)	0·1 (7)	0·10 (5)	0·2 (11)	0·2 (9)	0·04^4^
Nontyphoidal *Salmonella*^6^	0·5 (23)	0·02 (1)	0·04 (2)	0·3 (12)	0·2 (8)	0·002^4^
*Haemophilus influenzae*^7^	0·3 (14)	0	0	0·02 (1)	0·3 (13)	0·19
*Klebsiella* spp. ^8^	0·2 (12)	0·08 (4)	0·02 (1)	0·08 (4)	0·06 (3)	0·13
*Pseudomonas* spp.^9^	0·2 (9)	0·02 (1)	0	0·08 (4)	0·08 (4)	0·55
*Neisseria meningitidis*	0·12 (6)	0	0	0·04 (2)	0·08 (4)	0·30
*Enterobacter* spp. ^10^	0·10 (5)	0·08 (4)	0	0·02 (1)	0	0·83
Others	0·7 (36)	0·2 (11)	0·08 (4)	0·3 (12)	0·2 (9)	0·005^4^

1.Total live births within the actual DSS during the study period, 2001–2015, N = 47,651

2.Poisson regression assessing the incidence rate trend over time

3. One patient had both *S*. *aureus* and GAS isolated

4.p<0·05

5. One patient had both *E*. *coli* and *Klebsiella* spp. isolated

6. Includes *Salmonella* Typhimurium, *Salmonella* Enteritidis, *Salmonella* Heidelberg, *Salmonella* Isangi, and *Salmonella* spp.

7.Includes non-type b *H*. *influenzae*

8 Includes *K*. *pneumoniae*, *K*. *oxytoca*, *Klebsiella* spp.

9.Includes *P*. *aeruginosa*, *P*.*paucimobilis*, *Pseudomonas* spp.

10.Includes *E*. *cloacae*, *E*. *aglomerans*, *E*. *sakazakii*, *E*. *aerogenes*

Among the 57 young infants with invasive GBS disease, 19 had EOD and 38 had LOD (Table 1). GBS LOD incidence rates were equal to or higher than EOD incidence rates except for 2009, 2011 and 2014. Median age of onset was 10 days (EOD: day 0, LOD: day 13) and 24.6% (14/57) of GBS cases occurred during days 0–2, particularly on day 0 (10/57, 17.5%) (S1 Fig). CSF was collected in 42.1% of infants with GBS IBD; 62.5% of those were positive (71.4% for EOD, 58.8% for LOD). Mortality was higher among infants with EOD; approximately one-third of GBS EOD admissions resulted in death (Table 1).

A total of 35 GBS isolates (60% blood, 40% CSF; 88.6% from 2010 or later; 62.9% LOD) were available for characterization (Table 3, S4 Table). Almost all isolates (33 of 35, 94.3%) were serotype III. Serotype III isolates were all within clonal complex (CC) 17 (sequence type [ST] 17 and its single locus variants ST109, 866, and 1089), and were uniformly positive for the surface protein genes *hvgA*, *srr2*, *rib*, and for pilus subunit genes (PI1 and PI2b). The two unrelated non-serotype III isolates (serotype V/ST1 and serotype Ia/ST23) had different surface protein gene profiles, including the absence of the CC17-associated virulence factor (HvgA). All 35 isolates were resistant to tetracycline, corresponding to the presence of *tetM* gene (Table 3). Erythromycin resistance was identified in seven isolates; six were *mef-*positive (all serotype III) and one was *ermTR*-positive (serotype V) with inducible clindamycin resistance. Notably, all seven (21.9%) isolates with serotype III ST109 had a penicillin MIC of ≥0.12 μg/mL (five [15.6%] at 0.12 μg/mL, two [6.3%] at 0.25 μg/mL). All ST109 isolates had the unique PBP2x type PBP2x-39 (see ref [30] for the PBP2x typing scheme). Type PBP2x-39 contains three substitutions relative to the susceptible reference PBP2x transpeptidase domain sequence (I377V, G398A, G627V). It is likely that the G398A substitution confers the reduced susceptibility phenotype, since the other two substitutions are commonly found among basally beta-lactam susceptible GBS isolates[30]. These seven isolates were collected in different years (2005–2015) and each differed by 39–74 SNPs (S3 Fig).

**Table 3:** Microbiological and molecular characteristics of GBS isolates (n = 35).

Serotype	CC	ST	No. (%).	Specimen Type	Onset	Surface protein genes									PBP-2x type	Antibiotic resistance genes			Resistance phenotype^3^(no.)
						*hvgA*	*srr*		Pilus Island			Alpha family				Tetra-cycline	Lincosamides/macrolides		
				Blood (%)	LOD (%)		*1*	*2*	*1*	*2a1*	*2b*	*alp1*	*alp23*	*rib*		*tetM*	*mef*	*ermTR*	
IA	23	23	1 (2.9%)	100%	0%										5				
III	17	17	24 (68.6%)	66.7%	58.3%^1^										2				
																			Erythromycin (5)
	17 (slv)	109	7 (20.0%)	28.6%	71.4%^2^										39				
																			Penicillin (2)
																			Erythromycin (1)
		866	1 (2.9%)	100%	100%										2				
		1089	1 (2.9%)	0%	100%										2				
V	1	1	1 (2.9%)	100%	100%										1				Erythromycin, Inducible clindamycin (1)

All positive

1. 2 isolates with unknown day of onset

2. 1 isolate with unknown day of onset

3. All isolates were resistant to tetracycline (MIC ≥8 μg/ml). Erythromycin: erythromycin resistant (MIC ≥1 μg/ml); Inducible clindamycin: inducible clindamycin resistance present at erythromycin 1 μg/ml and clindamycin 0.5 μg/ml; Penicillin: reduced susceptibility to penicillin (MIC 0.25 μg/ml

CC = clonal complex. ST = sequence type. LOD = late onset disease. Slv = single locus variant. PBP = penicillin-binding protein.

## Discussion

GBS was a leading cause of IBD in Manhiça. About two-thirds of all GBS cases were LOD, the majority of GBS isolates were serotype III, and, though unexpected, there was molecular evidence of isolates with elevated MIC to penicillin. Although overall IBD incidence rates, neonatal mortality rates, and stillbirth rates decreased significantly during the study period, GBS rates were nearing a peak at the end of the study period, highlighting that the disease burden may not decrease without targeted interventions.

Pathogen-specific interventions that took place during this period included the introduction of Hib vaccine (2009) and PCV10 (2013). However, Hib IBD cases in this age group were already rare pre-vaccine and PCV10 introduction would not account for the observed IBD trend prior to 2013. Decrease in admission rates could have led to decreased IBD case ascertainment, but would not explain reductions in neonatal mortality and stillbirth rates in the community. Rather, the observed reductions in IBD, neonatal mortality, and stillbirth rates are likely due to other health interventions that took place during the study period, as well as general economic improvements in Mozambique: tetanus toxoid immunization contributed to elimination of maternal and neonatal tetanus nationwide in 2010[34], intermittent preventive treatment for malaria in pregnancy (IPTp) was introduced nationwide in 2006[35], and maternal access to HIV antiretroviral therapy increased from 34% in 2009 to 95% in 2015[36, 37]. Improvements in clean birth practices and access to appropriate interventions for sick neonates (e.g., anticonvulsants, antibiotics) may also have contributed[37].

Our results underestimate the actual GBS disease burden, particularly that of EOD. Between 80 to 90% of GBS EOD cases typically occur during the first 24 hours of life[38] and about two-thirds of young infant disease are EOD[10, 39]; however, day 0 cases were only 17.5% of all GBS cases in our study. In MDH, day 0–2 infants were less likely to have cultures collected compared to older infants. Thus, even with our established demographic and IBD surveillance systems, ascertainment of IBD in the first days of life remained challenging. Additionally, we may have missed GBS cases among infants who were transferred to the national referral hospital; however, the number of GBS cases among these infants is likely to be very small.

The predominance of serotype III GBS isolates may be due to the overrepresentation of LOD, as LOD has been associated with higher proportion of serotype III[10, 39]. Interestingly, the seven ST109 isolates had phenotypic evidence of elevated MIC to penicillin (MIC ≥0.12 μg/ml), supported by the presence of a new PBP2x type that contains a G398A substitution. It is known that amino acid substitutions closely adjacent to the conserved _402_SSN_404_ or _552_KSG_554_ motifs are often associated with reduced affinity for beta-lactam antibiotics[40, 41]. Epidemiologic data and phylogenic analyses of the seven ST109 isolates suggest this clone with elevated MIC to penicillin is well-established in the community and was not introduced by a point source. Historically, GBS has been pan-susceptible to beta-lactam antibiotics; however, emergence of GBS isolates with reduced beta-lactam susceptibility has raised concerns about the sustainability of penicillin or ampicillin as first-line antibiotics for IAP and treatment of infection[16]. Existing reports on GBS isolates with reduced susceptibility to penicillin have primarily been from Japan and North America among isolates from older age groups[41–43]. A recent U.S. study reported that 0.7% of the invasive GBS disease isolates had reduced susceptibility to penicillin (defined as MIC ≥0.25 μg/ml)[43], which is much lower than the finding in our study (6.3%), although our study was limited by the number of samples. A survey of 1975 invasive GBS disease isolates recovered in the United States during 2015 revealed only one isolate with reduced penicillin susceptibility[30], although 14 of the 1975 isolates (0.7%) contained first step *pbp2x* mutations conferring reduced susceptibility to beta-lactam antibiotics other than penicillin[30]. Molecular characterization of GBS isolates in sub-Saharan Africa has been rare[7] and, to our knowledge, this is the first molecular evidence of GBS isolates with reduced penicillin susceptibility in sub-Saharan Africa.

Our study is subject to limitations. First, there is a time-lag from when DSS events such as stillbirths and neonatal deaths occur to when they are captured and frequency of household visits by the DSS team decreased from twice a year to once a year in 2012. Thus, DSS event ascertainment may be lower in the more recent years. Second, the definition of IBD was based on a single positive culture result, so we may have included some cases based on isolates that were contaminants. Third, we did not have access to maternal data, in particular HIV status. Since exposure to HIV may increase the risk of LOD in particular[6, 44], maternal HIV status would provide a useful context for interpreting the high proportion of LOD we observed. Fourth, only a portion of GBS isolates were available for laboratory analyses. A larger sample size would allow for a more robust description of invasive disease strains. Lastly, our results are an underestimate of the true IBD burden, as described earlier.

Despite these limitations, we documented a notable young infant GBS disease burden despite reductions in overall IBD rate and broader improvements in maternal and neonatal health. In addition, this is the first report describing molecular characteristics of GBS isolates with reduced penicillin susceptibility in sub-Saharan Africa. Our findings that these isolates are established in this rural community and may be more prevalent in this region than previously considered, further underscore the need for non-antibiotic GBS prevention strategies such as maternal immunization.

## Supporting information

S1 Table Genomic sequence query coordinates for selected group B *Streptococcus* surface proteins. (DOCX)

S2 Table Invasive bacterial disease dataset and figures from Manhiça DSS. (XLSX)

S3 Table Main causes of death by age group based on verbal autopsy, 2001 to 2011. (DOCX)

S4 Table Whole genome sequencing biosample accessions for group B *Streptococcus* isolates. (XLSX)

S5 Table Clinical signs observed by the healthcare worker in young infants days 0–89 with IBD, Manhiça demographic and health surveillance system, Mozambique, 2001–2013. (DOCX)

S1 Fig Age distribution of young infants with invasive GBS, *S. pneumoniae* and *S. aureus* disease, Manhiça, 2001–2015. (DOCX)

S2 Fig **(a)Trends of incidence rates of gram positive pathogens in young infants (<90 days), Manhiça DSS, 2001–2015**.
GBS = group B *streptococcus*. S. aureus = *Staphylococcus aureus*. S. pneumo = *Streptococcus pneumoniae*. GDS = group D *streptococcus*.
**(b) Trends of incidence rates of Gram negative pathogens in young infants (<90 days), Manhiça DSS, 2001–2015**.
E.coli = *Escherichia coli*. NTS = Nontyphoidal *Salmonella*. H.flu = *Haemophilus influenzae*.
(DOCX)

S3 Fig Phylogenetic tree of serotype III isolates (n = 33). (DOCX)
