# Supplementary material for: Invasive bacterial disease trends and characterization of group B streptococcal isolates among young infants in southern Mozambique, 2001–2015
Source: PLoS One. 2018 Jan 19;13(1):e0191193. doi: 10.1371/journal.pone.0191193 (PMC5774717; doi:10.1371/journal.pone.0191193)
Supplement: S1 Table — (DOCX) [file pone.0191193.s001.docx]

**S1 Table. Genomic sequence query coordinates for selected group B *Streptococcus* surface proteins**

| **Protein** | **GenBank accession: query sequence coordinates (length in bp)** | **Features** |
| --- | --- | --- |
| HvgA | CP012480: 1958822-1959030 (209) | CC17-associated virulence factor, invasin |
| Alpha C family:   1. Alpha (Bca) 2. Rib 3. Alp1 4. Alp2-3 | 1. CP007570: 467645-468125 (510) 2. CP012480: 456093-456614 (522) 3. AY461799: 166-681 (516) 4. CP012503: 446508-447127 (620) | colonization, adhesion, invasion, antiphagocytic . Mutually exclusive and map at same genomic location |
| Serine rich repeat adhesins:   1. SRR1 2. SRR2 | 1. CP007570: 1498681-1498740 (60) 2. CP012480: 1379705-1379764 (60) | Colonization, adhesion, invasion |
| Pili:   1. PI1 2. PI2a1 3. PI2a2 4. PI2b | 1. CP012503: 749680-749739 (60) 2. CP012503: 1479525-1479466 (60) 3. CP019979: 1408669-1408728 (60) 4. CP012480: 1411739-1411798 (60) | colonization, adhesion, invasion, antiphagocytic . Most strains carry 1-2 of these pili. |
