# Supplementary material for: Invasive bacterial disease trends and characterization of group B streptococcal isolates among young infants in southern Mozambique, 2001–2015
Source: PLoS One. 2018 Jan 19;13(1):e0191193. doi: 10.1371/journal.pone.0191193 (PMC5774717; doi:10.1371/journal.pone.0191193)
Supplement: S3 Table — (DOCX) [file pone.0191193.s003.docx]

**S3 Table. Main causes of death by age group based on verbal autopsy, 2001 to 2011**

| **Day0***  **(n = 217)** | | **Day<7***  **(n = 468)** | | **Day<28***  **(n = 614)** | | **Day<90***  **(n = 881)** | |
| --- | --- | --- | --- | --- | --- | --- | --- |
| **Cause of death (block-ICD10)** | **No (%)** | **Cause of death (block-ICD10)** | **No (%)** | **Cause of death (block-ICD10)** | **No (%)** | **Cause of death (block-ICD10)** | **No (%)** |
| Respiratory and cardiovascular disorders specific to the perinatal period (P20-29) | 91 (41·9%) | Respiratory and cardiovascular disorders specific to the perinatal period (P20-29) | 160 (34·2%) | Respiratory and cardiovascular disorders specific to the perinatal period (P20-29) | 168 (27·4%) | Respiratory and cardiovascular disorders specific to the perinatal period (P20-29) | 174 (19·8%) |
| Disorders related to length of gestation and fetal growth (P05-08) | 40 (18·4%) | Disorders related to length of gestation and fetal growth (P05-08) | 99 (21·2%) | Disorders related to length of gestation and fetal growth (P05-08) | 124 (20·2%) | Disorders related to length of gestation and fetal growth (P05-08) | 128 (14·5%) |
| Other disorders originating in perinatal period (P90–P96) | 26 (12·0%) | Infection specific to the perinatal period (P35–39) | 61 (13·0%) | Infection specific to the perinatal period (P35–39) | 105 (17·1%) | Infection specific to the perinatal period (P35–39) | 114 (12·9%) |
| Infection specific to the perinatal period (P35–39) | 6 (2·8%) | Other disorders originating in perinatal period (P90–P96) | 28 (6·0%) | Other disorders originating in perinatal period (P90–P96) | 28 (4·6%) | Influenza and pneumonia (J10-J18) | 70 (7·9%) |
| Fetus and newborn affected by maternal factors and by complications of pregnancy, labor and delivery (P00–04) | 5 (2·3%) | Fetus and newborn affected by maternal factors and by complications of pregnancy, labor and delivery (P00–04)) | 8 (1·7%) | Influenza and pneumonia (J10-J18) | 14 (2·3%) | Malaria (B50 –54) | 41 (4·7%) |

*Age groups are not mutually exclusive and are cumulative
