# Supplementary material for: Invasive bacterial disease trends and characterization of group B streptococcal isolates among young infants in southern Mozambique, 2001–2015
Source: PLoS One. 2018 Jan 19;13(1):e0191193. doi: 10.1371/journal.pone.0191193 (PMC5774717; doi:10.1371/journal.pone.0191193)
Supplement: S5 Table — (DOCX) [file pone.0191193.s005.docx]

**S5 Table. Clinical signs observed by the healthcare worker in young infants days 0–89 with IBD,** **Manhiça Demographic and Health Surveillance System, Mozambique, 2001-2013^1^**

| **Characteristics** | **All IBD**  **N = 437**  **No (%)** | **All GBS**  **N = 57**  **No (%)** | **GBS** | |
| --- | --- | --- | --- | --- |
|  |  |  | **EOD**  **N = 19**  **no (%)** | **LOD**  **N = 38**  **no (%)** |
| Agitated or unconscious^2^ | 12 (2.8%) | 4 (7.0%) | 2 (10.5%) | 2 (5.3%) |
| Fever (≥38.0°C)^3^ | 159 (37.0%) | 18 (32.7%) | 6 (33.3%) | 12 (32.4%) |
| Low body temperature (<35.5°C)^3^ | 23 (5.4%) | 4 (7.3%) | 4 (21.1%) | 0 |
| Normal temperature^3^ | 248 (57.7%) | 33 (57.9%) | 8 (42.1%) | 25 (66.8%) |
| Tense fontanelle^4^ | 18 (4.1%) | 6 (10.5%) | 1 (5.3%) | 5 (13.2%) |
| Neck stiffness^5^ | 1 (0.2%) | 0 | 0 | 0 |
| Pallor^6^ | 33 (7.6%) | 7 (12.3%) | 5 (26.3%) | 2 (5.3%) |
| Jaundice^7^ | 26 (6.0%) | 3 (5.3%) | 0 | 3 (7.9%) |
| Chest indrawing | 144 (33.0%) | 22 (38.6%) | 8 (42.1%) | 14 (36.8%) |
| Fast breathing^8^ | 222 (51.4) | 29 (50.9%) | 7 (36.8%) | 22 (57.9%) |
| Nasal flaring^9^ | 115 (26.3) | 14 (24.6%) | 7 (36.8%) | 7 (18.4%) |
| Dehydration^10^ | 52 (11.9) | 6 (10.5%) | 2 (10.5%) | 4 (10.5%) |

EOD=early-onset disease. GBS=group B *streptococcus*. IBD=invasive bacterial disease. LOD=late-onset disease.

1. Percentage was calculated excluding those with missing data
2. 1 missing data (none among GBS cases)
3. 7 missing data on body temperature (2 among GBS cases: 1 EOD, 1 LOD)
4. 1 missing data (none among GBS cases)
5. 2 missing data (none among GBS cases)
6. 1 missing data (none among GBS cases)
7. 1 missing data (none among GBS cases)
8. 5 missing data (none among GBS cases)
9. 1 missing data (none among GBS cases)
10. 2 missing data (none among GBS cases)
