# Supplementary material for: Invasive bacterial disease trends and characterization of group B streptococcal isolates among young infants in southern Mozambique, 2001–2015
Source: PLoS One. 2018 Jan 19;13(1):e0191193. doi: 10.1371/journal.pone.0191193 (PMC5774717; doi:10.1371/journal.pone.0191193)
Supplement: S1 Fig — (DOCX) [file pone.0191193.s006.docx]

**S1 figure. Age distribution of young infants with invasive GBS, *S. pneumoniae* and *S. aureus* disease, Manhiça, 2001–2015**
