# Supplementary material for: Invasive bacterial disease trends and characterization of group B streptococcal isolates among young infants in southern Mozambique, 2001–2015
Source: PLoS One. 2018 Jan 19;13(1):e0191193. doi: 10.1371/journal.pone.0191193 (PMC5774717; doi:10.1371/journal.pone.0191193)
Supplement: S2 Fig — (a)Trends of incidence rates of gram positive pathogens in young infants (<90 days), Manhiça DSS, 2001–2015. GBS = group B streptococcus. S. aureus = Staphylococcus aureus. S. pneumo = Streptococcus pneumoniae. GDS = group D streptococcus. (b) Trends of incidence rates of Gram negative pathogens in young infants (<90 days), Manhiça DSS, 2001–2015. E.coli = Escherichia coli. NTS = Nontyphoidal Salmonella. H.flu = Haemophilus influenzae. (DOCX) [file pone.0191193.s007.docx]

**S2a figure. Trends of incidence rates of Gram positive pathogens in young infants (<90 days), Manhiça DSS, 2001–2015**

**S2b figure. Trends of incidence rates of Gram negative pathogens in young infants (<90 days), Manhiça DSS, 2001–2015**
