# Supplementary material for: Invasive bacterial disease trends and characterization of group B streptococcal isolates among young infants in southern Mozambique, 2001–2015
Source: PLoS One. 2018 Jan 19;13(1):e0191193. doi: 10.1371/journal.pone.0191193 (PMC5774717; doi:10.1371/journal.pone.0191193)
Supplement: S3 Fig — (DOCX) [file pone.0191193.s008.docx]

**S3 Figure. Phylogenetic tree of serotype III isolates (n = 33)**


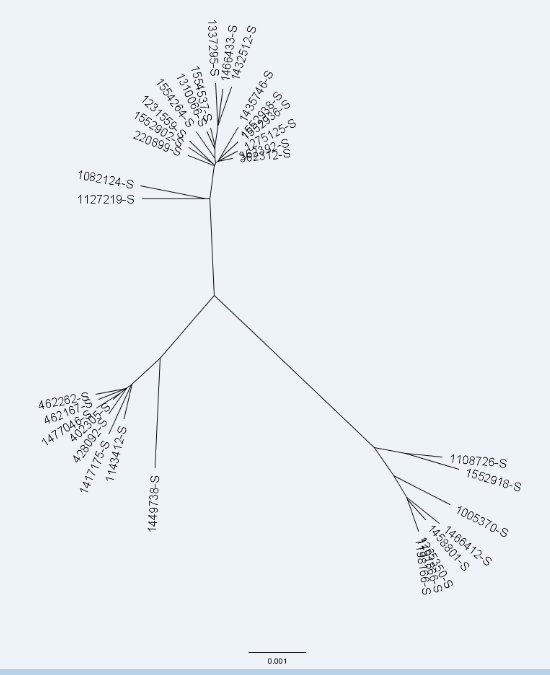


Core genome SNP identification and alignment were carried out using kSNP3.0.

The seven sequence type 109 isolates (circled in red) each differs by 39–74 single nucleotide polymorphisms
